# A Non-missile Orbitocranial Wooden Trajectile Extracted by Trans-conjunctival Approach: Exploring an Unconventional Avenue

**DOI:** 10.7759/cureus.76895

**Published:** 2025-01-04

**Authors:** Saroj Gupta, Rajamathangi Sunder, Fazal Khan, Radha Sarawagi, Jitendra Sharma

**Affiliations:** 1 Ophthalmology, All India Institute of Medical Sciences, Bhopal, IND; 2 Radiodiagnosis, All India Institute of Medical Sciences, Bhopal, IND

**Keywords:** orbit, ptosis, transconjunctival approach, trauma, wooden forgein body

## Abstract

A middle-aged female presented with drooping of the eyelid and loss of vision in her left eye (LE) following a fall at her residence 20 days back. On examination, in LE, vision was no light perception, with severe ptosis, ocular movements were restricted in all directions of gaze, and the fundus showed optic disc pallor. The right eye (RE) examination was normal. MRI of the brain and orbit showed a linear foreign body traversing between the medial and inferior recti, coursing through the superior orbital fissure close to the optic nerve. The posterior limit of the foreign body bordered on the left cavernous sinus. An urgent surgical extraction of the foreign body was done under general anaesthesia in conjunction with the neurosurgeon. A 7 cm long wooden twig was removed through a medial trans-conjunctival approach. This case report elaborates on the comprehensive management of an orbitocranial wooden foreign body in a middle-aged patient.

## Introduction

Trans-orbital penetrating injuries comprise 24% to 45% of the total penetrating head trauma among adults and children, respectively [1]. Based on the velocity of the projectile, they are classified as missiles and non-missiles. Non-missiles have an impact velocity of 100 m/s and cause injury via laceration, whereas missiles confound damage by kinetic and thermal energies [2]. Survival benefits with prompt interventions prove vital, albeit with a poor visual outcome. The authors present a case of left-sided penetrating orbital trauma caused by a 7 cm wooden foreign body (WFB) that extended from the superior orbital fissure to the cavernous sinus intracranially. The foreign body was successfully removed in its entirety using a transconjunctival approach.

## Case presentation

**Figure 1:**
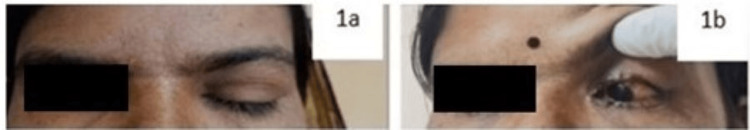
Images showing left eye ptosis. a and b: clinical photographs showing left eye ptosis with inferomedially rotated eyeball at presentation.

A middle-aged female presented in the outpatient services with complaints of redness, pain, and drooping of the eyelid associated with a gradual onset profound diminution of vision in the left eye since she suffered a fall at her residence 20 days back. There was a brief period of unconsciousness post-fall, followed by spontaneous recovery. She also noticed bleeding from the left nostril on bending down, which persisted for a few days post-trauma. At presentation, the visual acuity recorded was 20/20 in the right eye (RE) and absent perception of light in the left eye (LE). Ocular examination of LE revealed severe ptosis with absolute restriction of ocular movements in all gazes. The eyeball was displaced inferomedially (Figure 1a, Figure 1b).

Anterior segment examination showed conjunctival chemosis and congestion with a mid-dilated non-reacting pupil. A dilated fundus examination showed total optic disc pallor. RE examination was unremarkable. General examination was within normal limits with a Glasgow Coma Scale (GCS) of 15/15 at presentation.

**Figure 2:**
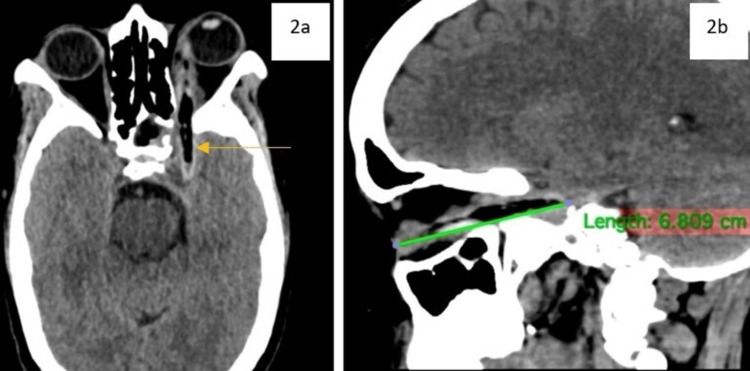
CT images. a and b: non-contrast computed tomography axial and sagittal view showing a linear hypodense foreign body of size 6.8 cm x 4.7 mm extending from the orbit to the left cavernous sinus passing through the superior orbital fissure.

Non-contrast computerized tomography of the brain with orbit axial and sagittal view showed a linear hypodense foreign body of size 6.8 cm x 4.7 mm (Figure 2a, Figure 2b) transversing through the left orbit (in-between the medial surface of the globe and medial wall of the orbit). Posteriorly, it was transversing through the superior orbital fissure (SOF) close to the optic nerve with no breach in the integrity of the globe. The posterior limit of the foreign body bordered on the left cavernous sinus with concomitant cavernous sinus thrombosis as suggested by heterogeneous hypodense areas in situ. The left sphenoid sinus showed mucosal thickness and was distended due to hemo-sinus.

**Figure 3:**
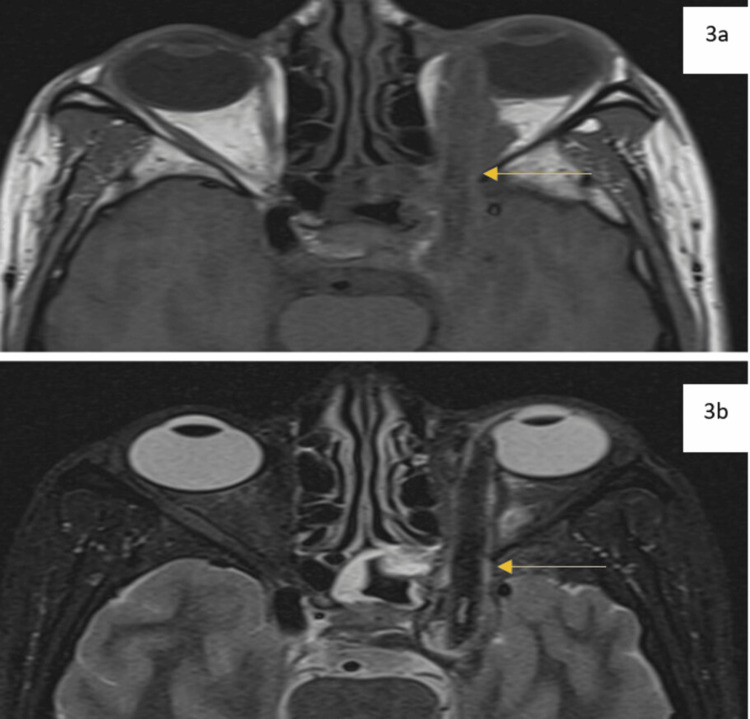
MRI images. a: T1 weighted magnetic resonance imaging reveals a wooden foreign body as a linear hypointense structure on the left side. (yellow arrow). b: T2 weighted magnetic resonance imaging reveals a hypointense foreign body with optic nerve compression at the orbital apex from granulation tissue (yellow arrow).

Further investigation with magnetic resonance imaging (MRI) displayed a linear T1, T2 hypointense structure of similar dimensions traversing between the medial and inferior recti, coursing through the superior orbital fissure, and terminating as aforementioned (Figure 3a, Figure 3b). The foreign body displaced the optic nerve superolateral with compression at the orbital apex from adjoining granulation tissue.

**Figure 4:**
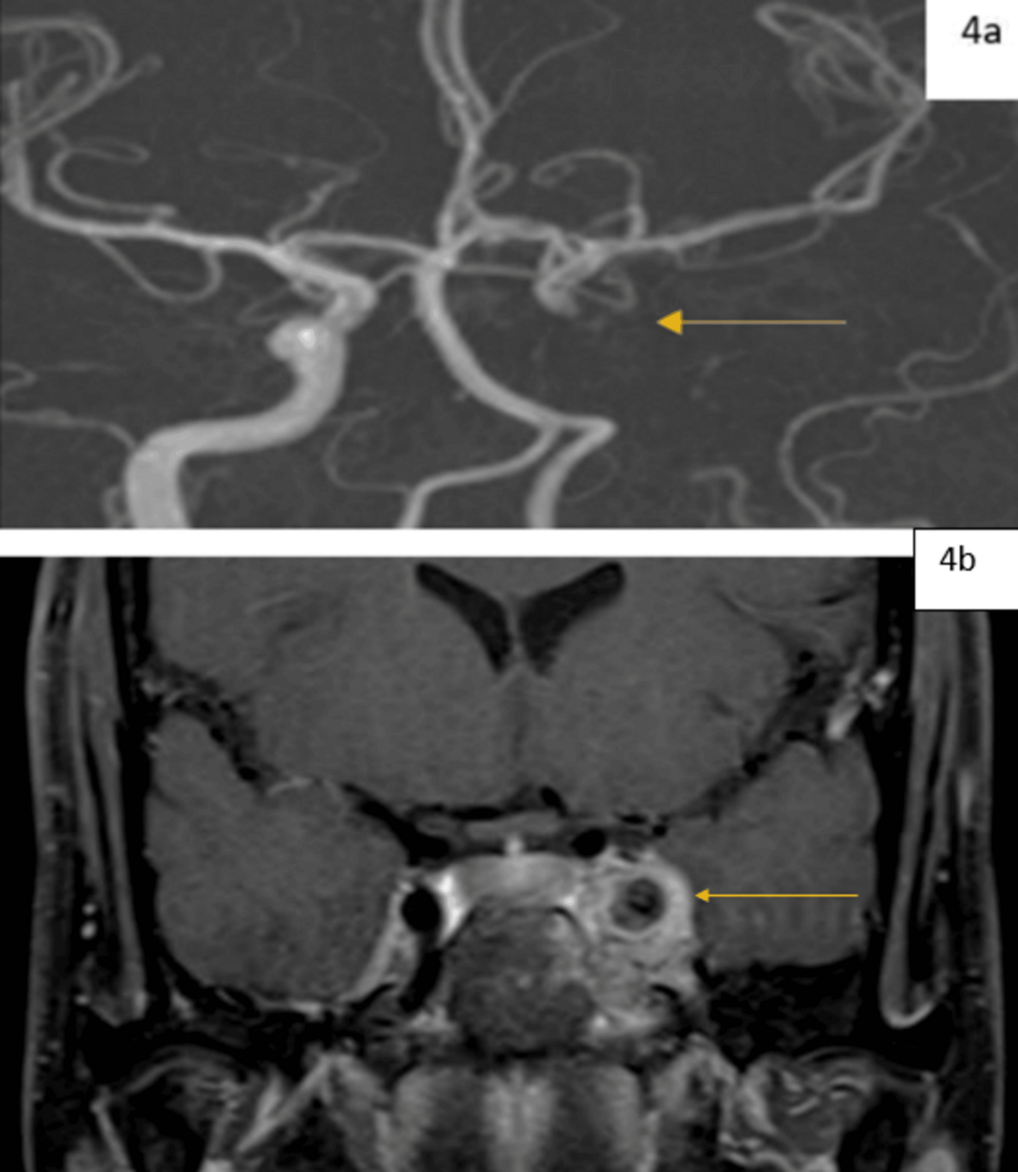
MR Angiograms. a: The MR angiogram reveals a loss of signal from the cavernous segment of the left internal carotid artery, which is suggestive of thrombosis (yellow arrow). b: T1 weighted post-contrast coronal image reveals a hypointense foreign body within the left cavernous sinus with heterogeneously enhancing granulation tissue (yellow arrow).

MR angiogram revealed a loss of signal from the cavernous segment of the left internal carotid artery, suggestive of thrombosis (Figure 4a). T1-weighted post-contrast coronal image revealed a hypointense foreign body within the left cavernous sinus with heterogeneously enhancing granulation tissue (Figure 4b).

In the context of the findings mentioned above, a clinical diagnosis of left-sided post-traumatic optic neuropathy with complete ophthalmoplegia inflicted by an orbito-cranial foreign body with super-added cavernous-sinus thrombosis was made. Other systemic examinations and lab investigations were unremarkable.

**Figure 5:**
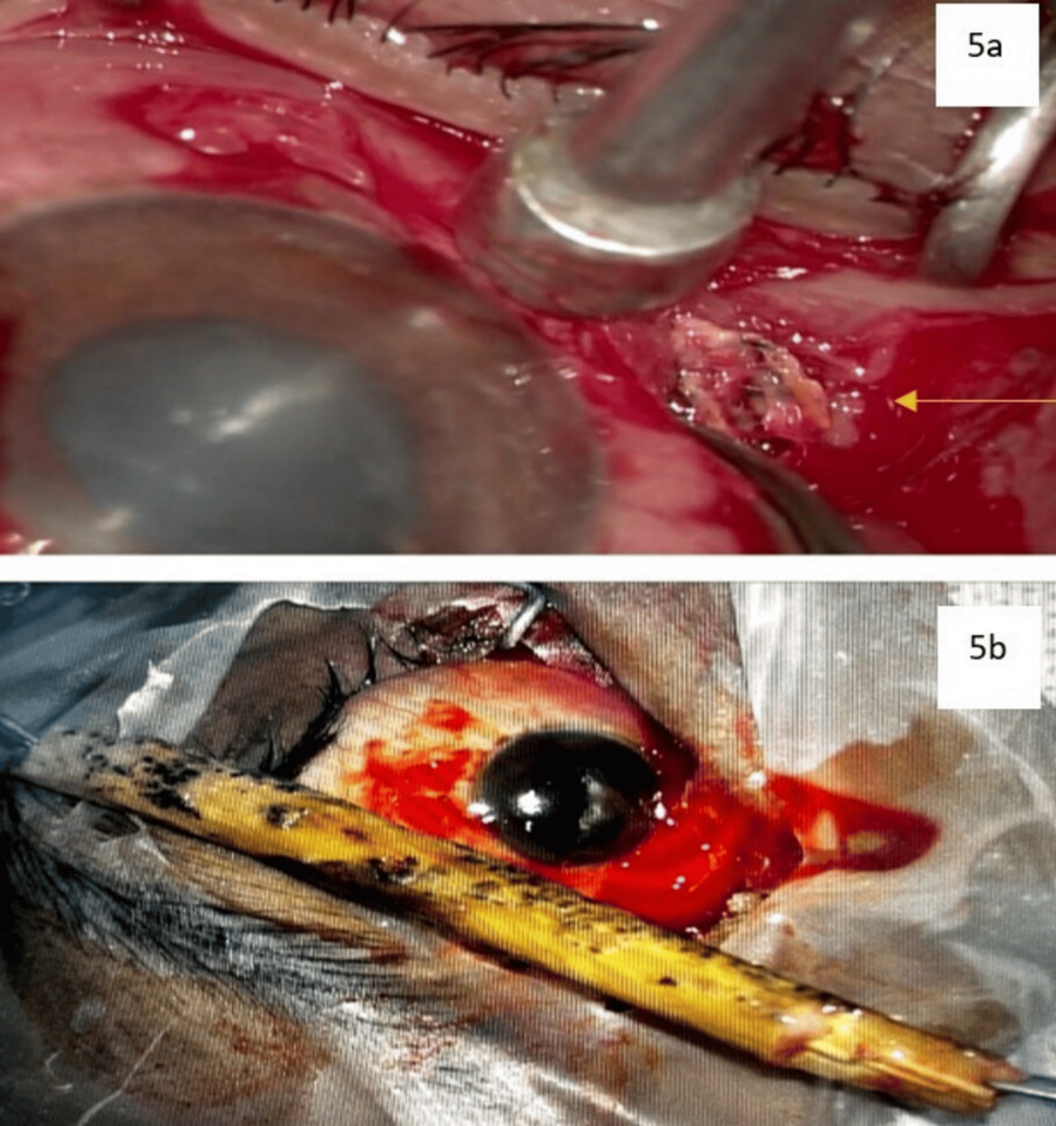
Intraoperative images. a: intra-operative image showing the deeply lodged wooden foreign body identified by trans-conjunctival approach. b: wooden foreign body post-retrieval in-toto.

An urgent surgical extraction of the foreign body was done under general anaesthesia in conjunction with the neurosurgery department under nil visual prognosis. A 7 cm long wooden twig was removed in-toto through a medial trans-conjunctival approach. The stump was identified from a deep exploration of periorbital tissue, and the foreign body was extricated in one piece with forceps (Figure 5a, Figure 5b).

Infiltration of the foreign-body track with vancomycin and ceftazidime, coupled with a thorough debridement of an early abscess appearing in the niche, prevented the intracranial ascent of infection. In the postoperative period, oral antibiotics (Tablet linezolid 600 mg BD for five days) and anti-epileptics (Tablet levetracetam 750 mg OD) were continued, and vitals were closely monitored.

During the postoperative follow-up, the patient remained under close supervision. Ptosis and ophthalmoplegia persisted in the follow-up period with nil visual recovery. However, conjunctival redness and chemosis decreased, and the fatal sequelae of an intracranially extending organic foreign body were intercepted with prompt interventions. The follow-up period was uneventful, with no episodes of fever or seizures.

## Discussion

We report a rare case of non-missile transorbital intracranial penetrating injury due to a wooden foreign body in a middle-aged female. With most trauma, the orbital apex, the optic canal, and the superior orbital fissure (SOF) are the usual channels giving way to the intracranial compartment. Fatal consequences are encountered when the trajectory courses through the SOF-cavernous sinus, terminating at the brainstem/cerebellum [3]. In our case, the wooden foreign body was traversing between the medial and inferior recti, coursing through the superior orbital fissure in close proximity to the optic nerve terminating at the cavernous sinus.

CT scan, MRI, and MR angiography are key imaging modalities used to determine the trajectory of the foreign body and the extent of brain tissue injury, to rule out any vascular injury, and in proper planning for surgery [3]. Pneumocephalus and dry wood appear identical on the CT scan except for the linear hypodense morphology of WFB. As the wood absorbs water, it appears similar to soft tissue, and hence, CT is not diagnostic in cases of organic foreign bodies. In contrast, MRI is a more sensitive imaging modality because a dry wooden foreign body appears hypointense on T1 and T2-weighted scans, as in our case. However, a wet wooden foreign body is hypointense on T1-weighted scans and hyperintense on T2-weighted scans [4]. Angiography is necessary for orbitocranial penetrating injuries to exclude cerebrovascular injuries and to delineate the relationship between the foreign body and the vascular structures [5]. In our case, the MR angiogram revealed thrombosis of the left cavernous sinus and supra-clinoid segment of the left internal carotid artery and superior ophthalmic vein.

Wooden foreign bodies are good culture media for microbial growth and increase the risk of orbital and intracranial infections. Therefore, timely and complete removal of WFB is mandatory [6]. Depending on the location of the foreign body, transcranial or transorbital approaches have been used for the removal of the orbitocranial foreign body. In previously published cases, transcranial, transorbital, or combined routes of retrieval have been used (Table 1) [1,3,6-11].

**Table 1:** Summary of selected published cases of orbito-cranial wooden foreign-body. SOF: superior orbital fissure, PL: perception of light.

	Authors, year	Age/gender	Presentation (visual acuity, clinical features )	Entry wound and trajectory	Dimensions of foreign body	Approach	Outcomes
1	Yamazaki et al. 2023 [1]	24/M	Counting fingers restricted ocular movements	Left lower lid, SOF	3.2 cm long, wood chopstick	Fronto-temporal craniotomy	20/20, left trochlear nerve palsy persisted.
2	Avraham et al. 2020 [3]	71/M	No PL, left periorbital edema, complete ophthalmoplegia, optic disc pallor	Left medial-canthus, SOF	3 wooden fragments, 3 cm each.	Trans-orbital with sub-temporal intra-dural approach.	No PL, complete ophthalmoplegia, Disc pallor
3	Dudani et al. 2019 [6]	7/M	No PL, proptosis, complete ophthalmoplegia	Right upper eyelid, SOF	9 cm x 1 cm wooden foreign body	Trans-orbital (supra-orbital space)	NO PL, Optic disc pallor
4	Parajuli et al. 2015 [7]	14/F	No PL, fixed non-reacting pupil, frozen globe, proptosis, optic disc edema, and flame-shaped hemorrhages.	Left inferior fornix, SOF	5 cm bamboo	Fronto-temporal craniotomy with transorbital approach	No PL, frozen eyeball.
5	Borkar et al. 2014 [8]	10/F	No PL, right globe perforation	Right medial-canthus, SOF	Wooden foreign body	Fronto-temporal craniotomy with evisceration	Post evisceration
6	Mohapatra et al. 2019 [9]	55/M	Preserved vision, CSF leak, aggressive behavior.	Right upper lid, orbital roof	Bamboo	Self-removal	Sensorium improved, and CSF leak sealed
7	Singh et al. 2013 [10]	30/M	No PL, proptosis, complete ophthalmoplegia, hyphema	Left upper lid, SOF	11 cm x 0.8 cm bamboo stick	NA	NA
8	Lee et al.1996 [11]	58 /M	20/20, fixed anisocoric pupil, restricted movements	Right upper lid, SOF	7.5 cm x 8 mm Wooden stick	Fronto-temporal craniotomy	Eyeball movements fully recovered.
9	Our case	35/F	No PL, frozen eyeball, fixed mid-dilated pupil, Optic disc pallor	left medial-canthus, SOF	6.8 cm x 4.7 mm wooden twig	Trans-conjunctival approach	No PL, frozen eyeball.

In our case, the transconjunctival approach was used, as the wound of the entry was visible near the medial canthus. On deep exploration, the stump of a foreign body was identified. A 7 cm long wooden twig was removed in-toto in conjunction with a neurosurgeon under general anaesthesia.

To elucidate, orbitocranial penetrating injury with a wooden foreign body is rare. There are a few case reports with large WFB with dimensions more than 7 cm X 6.1 mm [10,11]. Our case stands unique in the perspective of successful isolated trans-conjunctival retrieval of a large organic foreign-body in-toto without supplemental craniotomy for the intracranial fragment of the WFB. The trans-conjunctival route seems to be an unventured avenue. It is less invasive than craniotomy, with the added advantages of faster post-op recovery and better cosmesis. Early surgical exploration by a multidisciplinary team approach is essential to attain good recovery and a favourable outcome.

## Conclusions

In transorbital penetrating trauma, clinicians must be alert to intracranial communications and the extension of the foreign body, especially in non-missile projectiles. MRI is superior to CT scans in delineating the precise dimensions and location of an organic foreign body. Familiarization with the appearance of a dry and wet wooden foreign body on MRI-T1 and T2 scans is essential. Inter-departmental consultation with prior clarifications on the approach and technique prevents failure and misadventures.
